# Diagnostic Utility and Tendency of Bronchial and Serum Soluble Receptor for Advanced Glycation EndProducts (sRAGE) in Lung Cancer

**DOI:** 10.3390/cancers15102819

**Published:** 2023-05-18

**Authors:** Taehee Kim, Soo Jung Kim, Hayoung Choi, Tae Rim Shin, Yun Su Sim

**Affiliations:** 1 Division of Pulmonary, Allergy and Critical Care Medicine, Department of Internal Medicine, Hallym University Kangnam Sacred Heart Hospital, Seoul 07440, Republic of Korea; 2 Lung Research Institute, Hallym University College of Medicine, Chuncheon 24253, Republic of Korea

**Keywords:** receptor for advanced glycation end-products, lung cancer, biomarker

## Abstract

**Simple Summary:**

This study aimed to assess the diagnostic usefulness of serum receptor for advanced glycation end-products (sRAGE) levels in differentiating between infectious lung diseases and lung cancer. Serum and bronchial washing fluid samples were collected from patients with tuberculosis, pneumonia, or lung cancer, and sRAGE levels were measured using an enzyme-linked immunosorbent assay. The study found that while there was no significant difference in serum sRAGE levels between the groups, bronchial sRAGE levels were significantly different, with lung cancer showing the lowest levels. The results suggest that bronchial sRAGE levels could be used as an auxiliary diagnostic biomarker for lung cancer. The study concludes that bronchial sRAGE has potential as an adjuvant tool for diagnosis in tuberculosis, pneumonia, and lung cancer.

**Abstract:**

The receptor for advanced glycation end-products (RAGE) may serve as a diagnostic and prognostic biomarker of lung cancer and lung injury. We explored whether the serum and bronchial levels of soluble RAGE (sRAGE) distinguished infectious lung diseases from lung cancer. We collected serum and bronchial washing fluids (BWFs) from patients diagnosed with pneumonia, tuberculosis, or preoperative lung cancer from April 2016 to March 2022. sRAGE levels were measured using an enzyme-linked immunosorbent assay and we drew receiver operating characteristic (1) curves to determine the cut-off values affording the best diagnostic sensitivities. We enrolled 81 patients including 20 with tuberculosis, 30 with pneumonia, and 31 with lung cancer. Of the 81, 61% were males and the median age was 66 years. The median serum level of sRAGE was 822 (678–1168 pg/mL) and did not differ significantly between the three groups. The median bronchial sRAGE level was 167 (83–529 pg/mL) but 231 (108–649 pg/mL) for tuberculosis, 366 (106–706 pg/mL) for pneumonia, and 103 (32–254 pg/mL) for lung cancer patients (*p* = 0.018). The ROC curve for the bronchial sRAGE values of lung cancer patients revealed that the optimal cut-off was 118.9 pg/mL. This afforded a sensitivity of 76%, a specificity of 58%, and an area under the ROC curve of 0.695 (*p* = 0.005). The level of bronchial sRAGE differed significantly between patients with lung cancer and other respiratory diseases; that level may serve as an auxiliary diagnostic biomarker.

## 1. Introduction

The receptor for advanced glycation end-products (RAGE) is a transmembrane protein that is highly expressed in the type 1 alveolar epithelial cells that contribute to lung homeostasis [1]. RAGE, a member of the immunoglobulin superfamily, is a multiligand pattern-recognition receptor that binds advanced glycation end-products (AGEs), S100 proteins, and the amyloid β-peptide [2]. RAGE is activated by tissue injury or inflammatory processes [2]. Soluble RAGE (sRAGE) features an extracellular cytosolic domain that inhibits RAGE–ligand interactions by acting as a decoy that suppresses post-RAGE inflammatory signaling [3]. The sRAGE level is useful for diagnosing acute respiratory distress syndrome (ARDS) and is associated with both lung injury severity and prognosis [4,5]. RAGE plays an important role in lung homeostasis, and its water-soluble form, sRAGE, has shown promise as a diagnostic tool for lung inflammation.

In recent decades, a group of tumor-associated genes have been identified as potential regulators of carcinogenesis. Of particular importance is the interaction of RAGE, which may be involved in the development and progression of several types of cancer by inducing the generation of oxidative stress, and consequently, proliferation, angiogenesis, and inflammatory responses [6].

On the other hand, RAGE is associated with metastasis and poor prognosis in various types of cancer and at the same time RAGE levels may aid cancer diagnosis or predict prognosis [7,8,9,10]; studies on colorectal cancer [7] and oral squamous cell carcinoma [8] have reported RAGE upregulation. However, RAGE downregulation may be related to the prognosis of breast cancer [9,11] and the diagnosis of lung cancer [10,12].

Lung cancer is the second most frequent malignancy and the most lethal cancer worldwide [13]. Accurate diagnosis is essential in terms of both treatment and prognosis; various nonmalignant lesions including tuberculosis and pneumonia can be mistaken for lung cancer [14]. Tissue biopsy is most often used to confirm or exclude a malignant lesion. However, biopsy issues include bleeding, pneumothorax, air embolisms, and cancer seeding [15]. Clinical indicators of whether biopsy is required would be helpful. Therefore, we compared the diagnostic utilities of the serum and bronchial levels of sRAGE in patients with tuberculosis, pneumonia, and lung cancer, which usually can be difficult to differentiate clinically.

## 2. Materials and Methods

### 2.1. Study Design

The study was performed at a university teaching hospital between April 2016 and March 2022. Patients who underwent bronchoscopy and bronchial washing for infectious lung diseases (pneumonia, and pulmonary tuberculosis) and suspected lung cancer were enrolled. Microbiologic examination (Gram stain culture, AFB, TB-PCR, and fungal culture) and cytology were performed on bronchial washing specimens collected from all patients. The disease groups of pneumonia, tuberculosis, and lung cancer were defined according to the following clinical diagnostic criteria. Pneumonia was diagnosed on the basis of respiratory symptoms (cough, sputum, or chest tightness), clinical evidence of infection (fever, chills, or leukocytosis), and new or changed infiltrates as determined via chest radiography. Tuberculosis was diagnosed when an acid-fast bacillus culture was positive or when there was no response to broad-spectrum antibiotics and the imaging features were typical. Lung cancer was confirmed by the presence of malignant cells in pleural fluid, biopsy specimens, or bronchial lavage fluid and was staged using the International Association for the Study of Lung Cancer Staging Manual for Thoracic Oncology, edition 8 [16]. 

### 2.2. Bronchoscopy Procedures and Specimen Preparation

All bronchoscopic procedures were performed by four experienced pulmonologists using various video bronchoscopes (models BF-1T200, BF-260, BF-Q290, and BF-UC260FW; Olympus; Tokyo, Japan) via the nasal or oral route. Patients received local anesthesia (2% lidocaine spray) and mild conscious sedation with midazolam, with additional doses administered as needed during the procedure. After visualizing the vocal cords, the physician examined the trachea and bronchi, including the subsegmental bronchi. The selection of the subsegmental bronchi to be examined was performed by targeting the location of the lesion on the chest computed tomography. Bronchial washing fluid (BWF) collection was performed prior to biopsy. The target lesion or segmental bronchus to be examined was injected with 10–20 mL of sterile saline through the working channel in several divided doses, and a total of 40 mL of solution was aspirated, of which 5 mL was centrifuged for sRAGE immunoassay. The samples obtained through bronchial washing were spread onto multiple clean slides and immediately fixed in 95% ethyl alcohol. Papanicolaou stain was used to stain all the slides, which were then subjected to cytological evaluation, after being centrifuged for 5 min at 2000 rpm and preserved in 95% ethyl alcohol. The cell concentrates within the specimens were stained with Papanicolaou stain, while cellblock sections were stained with hematoxylin and eosin, following the standard protocol of the pathology laboratory. Cytopathologists interpreted all the slides, with the patients’ clinical information. Positive cytological results that clearly indicated a diagnosis of lung cancer were distinguished from negative results, which included non-diagnostic results or those showing benign, atypical, or suspicious cells without a definitive diagnosis.

### 2.3. Blood Collection

Blood samples were collected in at least 5 mL serum separator tubes, clotted for 30 min at room temperature, and centrifuged at 1000× *g* for 15 min. All blood samples were analyzed immediately or stored at ≤−20 °C.

### 2.4. Variables

BWF and peripheral blood were collected from patients with pneumonia, tuberculosis, or preoperative lung cancer. We centrifuged 5 mL each of BWF and peripheral blood samples and stored the supernatant at −70 °C. We retrieved data on age, sex, and comorbidities. Laboratory analysis was performed at the time of study enrollment and included white blood cell, neutrophil, and lymphocyte counts; the erythrocyte sedimentation rate (ESR); and the levels of C-reactive protein (CRP), procalcitonin, and carcino-embryonic antigen (CEA). 

### 2.5. Biochemical Assays

The quantitative determination of sRAGE concentrations in blood and BWF was performed using a commercial human sRAGE immuno-assay kit (R&D System, Minneapolis, MN, USA) according to manufacturer’s instructions. All the sRAGE levels in the serum and BWF were confirmed via duplicate analysis.

### 2.6. Ethical Statement

This study received ethical approval (Institutional Review Board no. 2016-03-016-002) from the Research Ethics Committee of Hallym University Kangnam Sacred Heart Hospital, and written informed consent was obtained from all patients. 

### 2.7. Statistical Analysis

Frequencies are expressed as numbers (%) and descriptive data are given as medians (interquartile ranges [IQRs]). The Chi-square test was used to compare categorical variables and the Kruskal–Wallis test was used to compare continuous variables. To determine the discriminatory powers of various bronchial sRAGE level cutoffs, we drew a receiver operating characteristic curve and calculated the area under the curve. All statistical analyses employed SPSS software ver. 18 and a *p*-value < 0.05 was chosen to indicate statistical significance.

## 3. Results

### 3.1. Clinical Characteristics

Eighty-one patients with pneumonia, tuberculosis, and lung cancer who had undergone bronchoscopy that yielded BWF between April 2016 and March 2022 were enrolled. Their clinical characteristics are presented in Table 1. There were 20 tuberculosis, 30 pneumonia, and 31 lung cancer patients. Males constituted 61% of the sample and the median age was 66 years. There was no among-group difference in sex or age. The most common clinical symptoms were fever, observed in 25% of tuberculosis patients and sputum, observed in 33% of pneumonia patients. Of the lung cancers, 90% were non-small cell lung cancers (45% adenocarcinomas and 45% squamous cell carcinomas). Stage 4 was the most common stage (51.6% of patients).

### 3.2. sRAGE Levels in Serum and BWF

The laboratory data are shown in Table 2. 

**Figure 1:**
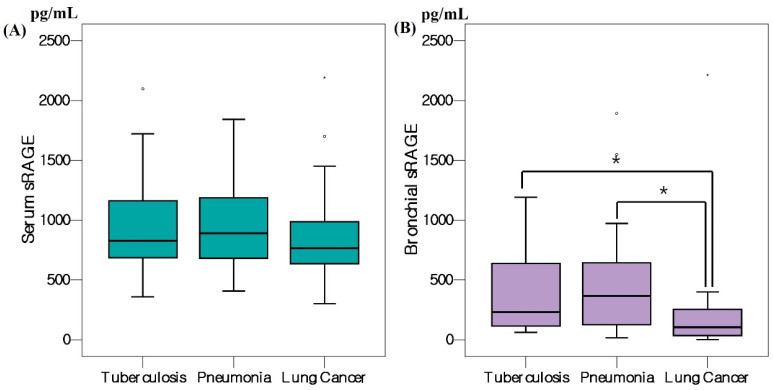
Boxplots of (**A**) serum and (**B**) bronchial sRAGE levels by the diagnoses. * *p* < 0.05.

The serum and BWF levels of sRAGE are shown in Table 2 and Figure 1. 

**Figure 2:**
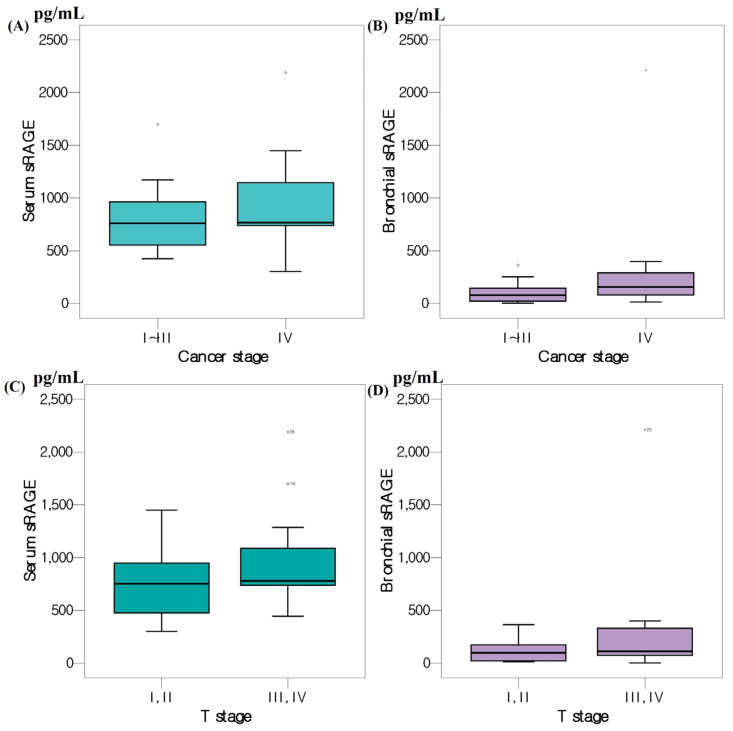
Boxplots of serum and bronchial sRAGE levels by the type of lung cancer (**A**,**B**) and by the stage of lung cancer (**C**,**D**).

The median serum sRAGE level was 822 (678–1168 pg/mL) but 828 (679–1195 pg/mL), 889 (680–1208 pg/mL), and 766 (587–1004 pg/mL) in tuberculosis, pneumonia, and lung cancer patients, respectively. It was slightly lower in lung cancer patients than others but not significant. Serum level of sRAGE was slightly higher in adenocarcinoma than squamous cell carcinoma patients but not significant. (Figure 2). The median bronchial sRAGE level was 167 (83–529 pg/mL) but 231 (108–649) in tuberculosis, 366 (106–706) in pneumonia, and 103 (32–254 pg/mL) in lung cancer patients. Bronchial level of sRAGE was slightly higher in Stage 4 lung cancer patients than in Stages 1 to 3 but not significant (Figure 2).

In the cytology results, the serum sRAGE and bronchial sRAGE levels were compared between the cancer cell positive and negative groups. The level of serum sRAGE was lower in the group that was cancel cell positive in cytology test (733 (442–794 pg/mL) vs. 818 (713–1152 pg/mL)), but it was not statistically significant (*p* = 0.057), and the bronchial sRAGE level was statistically different from the cancel cell positive in cytology group (92 (35–208 pg/mL) vs. 111 (20–271 pg/mL), *p* = 0.979). (Supplement Figure S1A,B).

The levels of serum and bronchial sRAGE were compared by creating two groups according to the stages: the T-stage I and II groups and the T-stage III and IV groups; the levels of each sRAGE in the two groups showed no statistically significant difference (Supplement Figure S1C,D).

**Figure 3:**
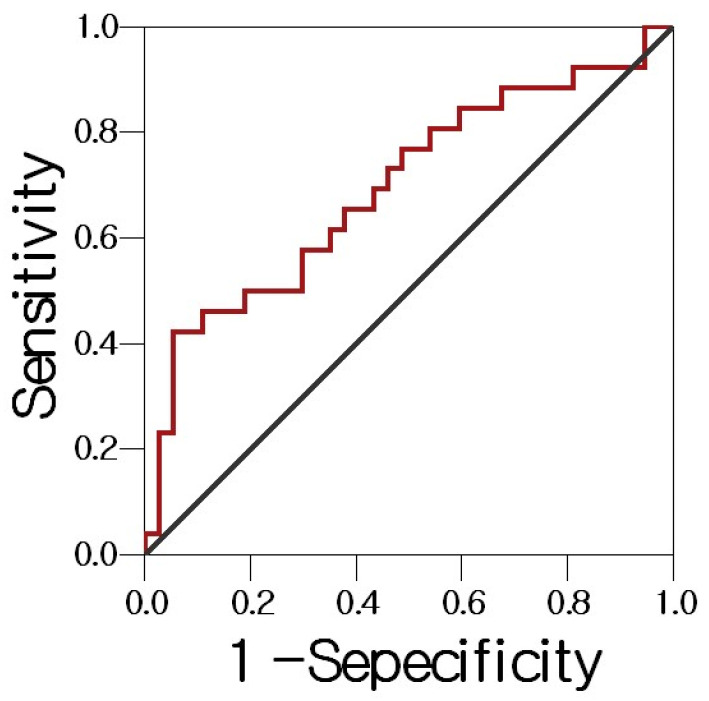
Receiver operating characteristic curve of bronchial sRAGE levels in patients with lung cancer and other conditions. The AUC is 0.710 (95% confidence interval, 0.579–0.841, *p* = 0.005).

The ROC curve showed that a bronchial sRAGE cutoff of 118.9 pg/mL afforded the best sensitivity (76%), specificity (58%), and AUC (0.695; *p* = 0.005) (Figure 3).

## 4. Discussion

The serum level of sRAGE did not significantly differ among patients with tuberculosis, pneumonia, and lung cancer but the bronchial sRAGE level was significantly lower in lung cancer patients than in others. At a bronchial sRAGE cutoff of 118.9 pg/mL, lung cancer was diagnosed with a sensitivity of 76% and a specificity of 58%. In a previous study [10], the serum level of sRAGE in lung cancer patients was significantly lower than in healthy controls or tuberculosis patients. In another study that analyzed sRAGE levels in the bronchoalveolar lavage (BAL) fluids of patients with various lung diseases [17], the sRAGE level was lower than that in patients with respiratory infections, but the proportion of lung cancer patients was small.

Pneumonia and tuberculosis are associated with lung inflammation; serum and bronchial sRAGE levels of people diagnosed with pneumonia and tuberculosis are higher than those of healthy controls [17,18]. It is unclear why the sRAGE level decreases in lung cancer patients. Binding of RAGE to AGEs induces the production of cytokines and growth factors, which can trigger an inflammatory response that causes cancer, whereas an increase in sRAGE blocks intracellular signal transduction by AGEs, preventing inflammation and oxidative stress that can cause cancer [19,20,21]. One animal study found that amphoterin binds to RAGE and that the establishment of a RAGE–amphoterin axis blockade in mice suppressed tumor cell migration and the production of tissue metalloproteinases [22]. Upregulation of sRAGE may impair the amphoterin- and RAGE-mediated stimulation of cancer growth and migration [22]. Studies on human alveolar epithelial A549 cells have suggested that the RAGE axis is involved in the repair of lung epithelial cells and perhaps also in cell migration and proliferation [23]. However, sRAGE level decreases in lung cancer patients [10,12,17] but increases in those with other types of cancer [7,8]. The roles played by sRAGE in cancer are complicated and remain to be elucidated. We found that the bronchial sRAGE levels were lower in lung cancer than in tuberculosis or pneumonia patients, but the serum levels did not significantly differ. The reasons why the bronchial but not the serum levels of sRAGE differ remain unclear. In a study on serum samples from 45 patients [10], the serum levels of sRAGE were significantly lower in lung cancer patients than in a control group. Although comparisons are difficult given the few other studies that have measured serum levels of sRAGE in lung cancer patients, some works have reported lower levels of RAGE in lung tissue [10,12] or BAL [17] of such patients than those with other diseases. The difference between the serum and bronchial levels of sRAGE may be explained as follows. RAGE is highly expressed in type 1 alveolar epithelial cells [1], and lung cancer initially develops in the lungs. Thus, changes in sRAGE levels in bronchial samples may occur before changes in serum. In patients with respiratory infections, such as tuberculosis and pneumonia, the serum and bronchial levels correlate because disease progression is rapid, but lung cancer progression is slow compared to that of a respiratory infection. Thus, changes in serum sRAGE will be later or lower than changes in bronchial sRAGE.

In studies that have investigated the expression levels of RAGE in tissues [10,12], expression was found to decrease as lung cancer progressed or lymph node metastasis developed, but we found no significant difference in serum or bronchial sRAGE levels by lung cancer stage or type. We also evaluated serum and bronchial sRAGE levels by metastasis location and pleural effusion status but found no significant difference. In the future, it may be useful to analyze the relationship between lung cancer progression or prognosis and serum or bronchial sRAGE levels in large-scale studies.

The strength of our work is that we are the first to comprehensively measure the levels of bronchial and serum sRAGE in lung cancer patients compared to those with tuberculosis and pneumonia. However, several limitations must be mentioned. First, our study had the inherent limitations of a case–control study, including recall and selection bias. Due to the limitations of the retrospective study, there was no definite case of pneumonia in tuberculosis and lung cancer patients, but the possibility of mild bronchitis or bacterial infection cannot be completely ruled out. Second, the small number of subjects for each disease made it difficult to specify the robustness of the statistical power and precision of our results and to sub-analyze by disease characteristics. Therefore, further larger sample studies are needed to validate the results of our study. Third, our study design compared sRAGE levels in lung disease groups (pneumonia, tuberculosis, and lung cancer) and did not include results for healthy controls without lung disease. Future studies should explore the differences in sRAGE levels between healthy control and lung disease groups. Fourth, BWF may be less informative than BAL. However, bronchial washing is minimally invasive, repeatable, and rarely causes complications.

We evaluated whether serum or bronchial sRAGE levels could serve as an adjuvant diagnostic tool before invasive procedures (such as biopsy) in patients with lung cancer and other diseases that can be mistaken for lung cancer, and the role played by sRAGE in the progression of respiratory disease. We found that serum levels did not significantly differ between lung cancer patients and those with other diseases, but bronchial levels were significantly lower, suggesting that they can serve as an auxiliary diagnostic tool. However, the present data and statistical analysis suggest that bronchial sRAGE can be used as an adjuvant marker in lung cancer diagnosis, but it is not definitive. In order to evaluate whether bronchial sRAGE is useful as an auxiliary diagnostic marker in lung cancer patients, it is necessary to conduct external validation [24] by preparing a validation cohort of a new patient group with lung disease at another institution, and not the patients in this study.

In addition, time-series analysis of changes in serum and bronchial sRAGE, from diagnosis to prognosis and prognosis of lung cancer, is expected to provide additional information about the role of RAGE in the pathophysiology of lung cancer. A serial study on serum and bronchial sRAGE in a lung cancer cohort may be needed in the future.

## 5. Conclusions

The level of bronchial sRAGE differed significantly between patients with lung cancer and other respiratory diseases. Bronchial sRAGE may serve as an auxiliary diagnostic biomarker. In order to evaluate the clinical usefulness of bronchial sRAGE as an adjuvant diagnostic marker in lung cancer patients, external validation and serial analysis in lung cancer cohorts will be needed in the future.

## Figures and Tables

**Table 1:** Clinical characteristics of patients.

	All Patients (*n* = 81)	Tuberculosis (*n* = 20)	Pneumonia (*n* = 30)	Lung Cancer (*n* = 31)	*p*-Value
Sex, male	49 (61%)	15 (75%)	16 (53%)	18 (58%)	0.289
Age, year	66 (54–76)	66 (49–74)	61 (−53–70)	73 (62–79)	0.034
BMI, kg/m^2^	23.0 (19.8–25.8)	22.7 (18.8–25.7)	22.5(19.4–25.8)	23.2 (20.6–25.8)	0.382
Smoking	32 (40%)	7 (35%)	13 (46%)	12 (37%)	0.599
Current smoker	17 (21%)	3 (15%)	5 (17%)	9 (29%)	0.372
Ex-smoker	15 (19%)	4 (20%)	8 (27%)	3 (10%)	0.228
Never-smoker	49 (61%)	13 (65%)	17 (57%)	19 (61%)	0.834
Amount, PY	35 (26–50)	2 (0–30)	0 (0–30)	30 (0–50)	0.202
Symptom					
Fever	10 (12%)	5 (25%)	5 (17%)	0	0.020
Sputum	13 (16%)	3 (15%)	10 (33%)	0	0.002
Cough	31 (39%)	7 (35%)	13 (43%)	11 (26%)	0.770
Hemoptysis	10 (12%)	2 (10%)	7 (23%)	1 (3%)	0.054
Comorbidity					
Hypertension	34 (42%)	7 (35%)	12 (405)	15 (48%)	0.615
Diabetes	17 (21%)	4 (20%)	4 (13%)	9 (29%)	0.320
COPD	9 (11%)	1 (5%)	4 (13%)	4 (13%)	0.604
Asthma	10 (12%)	2 (10%)	2 (7%)	6 (19%)	0.301
Cancer type					
NSCLC				28 (90%)	
ADC				14 (45%)	
SqCC				14 (45%)	
SCLC				2 (6.5%)	
Other cancer				1 (3.2%)	
Stage					
I				7 (22.6%)	
II				2 (6.5%)	
III				6 (19.4%)	
IV				16 (51.6%)	

Data for continuous and categorical variables are presented as median (interquartile ranges) and numbers (%), respectively. BMI, body mass index; PY, pack-year; COPD, chronic obstructive pulmonary disease; NSCLC, non-small cell lung cancer; ADC, adenocarcinoma; SqCC, squamous cell carcinoma; SCLC, small cell lung cancer.

**Table 2:** Laboratory characteristics of patients.

	All Patients (*n* = 81)	Tuberculosis (*n* = 20)	Pneumonia (*n* = 30)	Lung Cancer (*n* = 31)	*p*-Value
Serum sRAGE, pg/mL	822 (678–1168)	828 (679–1195)	889 (680–1208)	766 (587–1004)	0.561
BWF sRAGE, pg/mL	167 (83–529)	231 (108–649)	366 (106–706)	103 (32–254)	0.018
WBC, ×10^3^/m^3^	7.47 (5.87–9.70)	7.66 (5.87–9.76)	7.16 (5.67–11.8)	7.47 (5.90–9.23)	0.675
Neutrophil, %	68 (58–75)	68 (58–75)	68 (58–83)	67 (58–73)	0.320
Lymphocyte, %	21 (14–29)	21 (14–29)	21 (6.6–29)	2.2 (1.4–19)	0.1329
ESR, mm/hr	39 (14–65)	46 (13–66)	49 (21–76)	58 (11–57)	0.342
CRP, mg/dL	12.5 (1.7–81.6)	13.9 (1.9–95.4)	32.5 (1.5–118.1)	5.2 (1.6–31.6)	0.303
Procalcitonin, ng/dL	0.08 (0.03–0.21)	0.09 (0.02–0.33)	0.12 (0.035–0.39)	0.03 (0.03–0.10)	0.178
CEA, ng/mL	1.9 (1.6–6.0)	1.3 (0.9–1.8)	3.8 (1.6–6.0)	3.2 (1.7–10.0)	0.038
HbA1c, %	5.85 (5.40–6.33)	5.8 (5.6–6.0)	5.7 (5.4–6.4)	5.9 (5.6–6.4)	0.938
Tuberculosis cultured	15 (19%)	15 (75%)	0	0	<0.001
Glucose, mg/dL	112 (99–146)	113 (97–147)	113 (92–146)	112 (101–143)	0.892

Data are presented as medians (interquartile rage) or numbers (%). sRAGE, soluble receptor for advanced glycation end products; BWF, bronchial washing fluid; WBC, white blood cell; ESR, erythrocyte sedimentation rate; CRP, C-reactive protein; CEA, carcino-embryonic antigen; HbA1c, glycated hemoglobin.

## Data Availability

The data presented in this study are available on request from the corresponding author.

## Supplementary Materials

The following supporting information can be downloaded at: https://www.mdpi.com/article/10.3390/cancers15102819/s1, Figure S1: Boxplot of serum soluble receptor for advanced glycation end products (sRAGE) level in negative cytology (A), positive cytology (B) T stage I and II (C), and T stage III and IV (D).

## References

[1] Buckley S.T., Ehrhardt C. (2010). The receptor for advanced glycation end products (RAGE) and the lung. J. Biomed. Biotechnol..

[2] Stern D., Yan S.D., Yan S.F., Schmidt A.M. (2002). Receptor for advanced glycation endproducts: A multiligand receptor magnifying cell stress in diverse pathologic settings. Adv. Drug. Deliv. Rev..

[3] Raucci A., Cugusi S., Antonelli A., Barabino S.M., Monti L., Bierhaus A., Reiss K., Saftig P., Bianchi M.E. (2008). A soluble form of the receptor for advanced glycation endproducts (RAGE) is produced by proteolytic cleavage of the membrane-bound form by the sheddase a disintegrin and metalloprotease 10 (ADAM10). FASEB J..

[4] Calfee C.S., Ware L.B., Eisner M.D., Parsons P.E., Thompson B.T., Wickersham N., Matthay M.A., Network N.A. (2008). Plasma receptor for advanced glycation end products and clinical outcomes in acute lung injury. Thorax.

[5] Jabaudon M., Blondonnet R., Roszyk L., Bouvier D., Audard J., Clairefond G., Fournier M., Marceau G., Dechelotte P., Pereira B. (2015). Soluble Receptor for Advanced Glycation End-Products Predicts Impaired Alveolar Fluid Clearance in Acute Respiratory Distress Syndrome. Am. J. Respir. Crit. Care Med..

[6] Yamagishi S., Matsui T., Fukami K. (2015). Role of receptor for advanced glycation end products (RAGE) and its ligands in cancer risk. Rejuvenation Res..

[7] Kuniyasu H., Chihara Y., Takahashi T. (2003). Co-expression of receptor for advanced glycation end products and the ligand amphoterin associates closely with metastasis of colorectal cancer. Oncol. Rep..

[8] Sasahira T., Kirita T., Bhawal U.K., Yamamoto K., Ohmori H., Fujii K., Kuniyasu H. (2007). Receptor for advanced glycation end products (RAGE) is important in the prediction of recurrence in human oral squamous cell carcinoma. Histopathology.

[9] Tesarova P., Kalousova M., Jachymova M., Mestek O., Petruzelka L., Zima T. (2007). Receptor for advanced glycation end products (RAGE)--soluble form (sRAGE) and gene polymorphisms in patients with breast cancer. Cancer Investig..

[10] Jing R., Cui M., Wang J., Wang H. (2010). Receptor for advanced glycation end products (RAGE) soluble form (sRAGE): A new biomarker for lung cancer. Neoplasma.

[11] Peng Y., Liu F., Qiao Y., Wang P., Du H., Si C., Wang X., Chen K., Song F. (2022). Genetically Modified Circulating Levels of Advanced Glycation End-Products and Their Soluble Receptor (AGEs-RAGE Axis) with Risk and Mortality of Breast Cancer. Cancers.

[12] Bartling B., Hofmann H.S., Weigle B., Silber R.E., Simm A. (2005). Down-regulation of the receptor for advanced glycation end-products (RAGE) supports non-small cell lung carcinoma. Carcinogenesis.

[13] Siegel R.L., Miller K.D., Fuchs H.E., Jemal A. (2022). Cancer statistics, 2022. CA Cancer J. Clin..

[14] Neacsu F., Varban A.S., Simion G., Surghie R., Patrascu O.M., Sajin M., Dumitru M., Vrinceanu D. (2021). Lung cancer mimickers-a case series of seven patients and review of the literature. Rom. J. Morphol. Embryol..

[15] Wiener R.S., Wiener D.C., Gould M.K. (2013). Risks of Transthoracic Needle Biopsy: How High?. Clin. Pulm. Med..

[16] Detterbeck F.C. (2018). The eighth edition TNM stage classification for lung cancer: What does it mean on main street?. J. Thorac. Cardiovasc. Surg..

[17] Kamo T., Tasaka S., Tokuda Y., Suzuki S., Asakura T., Yagi K., Namkoong H., Ishii M., Hasegawa N., Betsuyaku T. (2015). Levels of Soluble Receptor for Advanced Glycation End Products in Bronchoalveolar Lavage Fluid in Patients with Various Inflammatory Lung Diseases. Clin. Med. Insights Circ. Respir. Pulm. Med..

[18] da Silva L.F., Skupien E.C., Lazzari T.K., Holler S.R., de Almeida E.G.C., Zampieri L.R., Coutinho S.E., Andrades M., Silva D.R. (2019). Advanced glycation end products (AGE) and receptor for AGE (RAGE) in patients with active tuberculosis, and their relationship between food intake and nutritional status. PLoS ONE.

[19] Ahmad S., Khan H., Siddiqui Z., Khan M.Y., Rehman S., Shahab U., Godovikova T., Silnikov V., Moinuddin (2018). AGEs, RAGEs and s-RAGE; friend or foe for cancer. Semin. Cancer Biol..

[20] Perrone A., Giovino A., Benny J., Martinelli F. (2020). Advanced Glycation End Products (AGEs): Biochemistry, Signaling, Analytical Methods, and Epigenetic Effects. Oxid. Med. Cell. Longev..

[21] Erusalimsky J.D. (2021). The use of the soluble receptor for advanced glycation-end products (sRAGE) as a potential biomarker of disease risk and adverse outcomes. Redox Biol..

[22] Taguchi A., Blood D.C., del Toro G., Canet A., Lee D.C., Qu W., Tanji N., Lu Y., Lalla E., Fu C. (2000). Blockade of RAGE-amphoterin signalling suppresses tumour growth and metastases. Nature.

[23] Zhai R., Blondonnet R., Ebrahimi E., Belville C., Audard J., Gross C., Choltus H., Henrioux F., Constantin J.M., Pereira B. (2020). The receptor for advanced glycation end-products enhances lung epithelial wound repair: An in vitro study. Exp. Cell Res..

[24] Ramspek C.L., Jager K.J., Dekker F.W., Zoccali C., van Diepen M. (2021). External validation of prognostic models: What, why, how, when and where?. Clin. Kidney J..

