# Supplementary figures and images for: Diagnostic Utility and Tendency of Bronchial and Serum Soluble Receptor for Advanced Glycation EndProducts (sRAGE) in Lung Cancer
Source: Cancers (Basel). 2023 May 18;15(10):2819. doi: 10.3390/cancers15102819 (PMC10216359; doi:10.3390/cancers15102819)

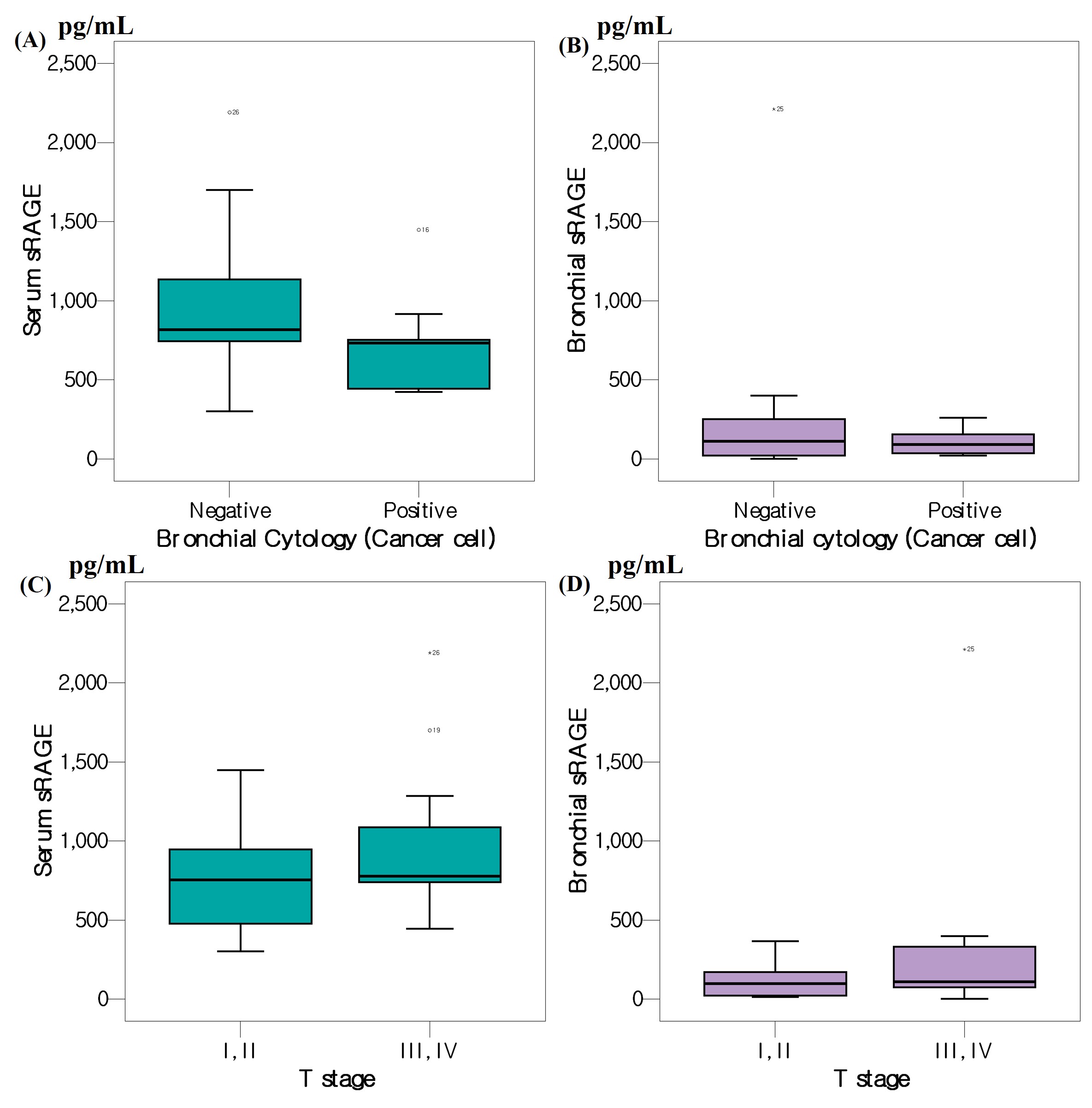

Supplement: Supplementary file 1 [file cancers-15-02819-s001.zip › cancers-2293974-Figure S1.jpg]
